# Supplementary material for: Self-assembly of tessellated tissue sheets by expansion and collision
Source: Nat Commun. 2022 Jul 12;13:4026. doi: 10.1038/s41467-022-31459-1 (PMC9276766; doi:10.1038/s41467-022-31459-1)
Supplement: Supplementary file 1 — Supplementary Information [file 41467_2022_31459_MOESM1_ESM.pdf]

SUPPLEMENTARY INFORMATION

Self-assembly of tessellated tissue sheets by growth and collision

Matthew A. Heinrich[1,*], Ricard Alert[2,3,4,5,*], Abraham E. Wolf[6,*], Andrej Košmrlj[1,7,‡], Daniel J. Cohen[1,6,‡]

(1) Department of Mechanical and Aerospace Engineering, Princeton University, Princeton, NJ 08544, USA

(2) Princeton Center for Theoretical Science, Princeton University, Princeton, NJ 08544, USA

(3) Lewis-Sigler Institute for Integrative Genomics, Princeton University, Princeton, NJ 08544, USA

(4) Max Planck Institute for the Physics of Complex Systems, Nöthnitzerst. 38, 01187 Dresden, Germany

(5) Center for Systems Biology Dresden, Pfotenhauerst. 108, 01307 Dresden, Germany

(6) Department of Chemical and Biological Engineering, Princeton University, Princeton, NJ 08544, USA

(7) Princeton Institute of Materials, Princeton University, Princeton, NJ 08544, USA

*These three authors contributed equally to this work.

‡Corresponding authors:
Andrej Košmrlj (andrej@princeton.edu) and Daniel J. Cohen (danielcohen@princeton.edu)

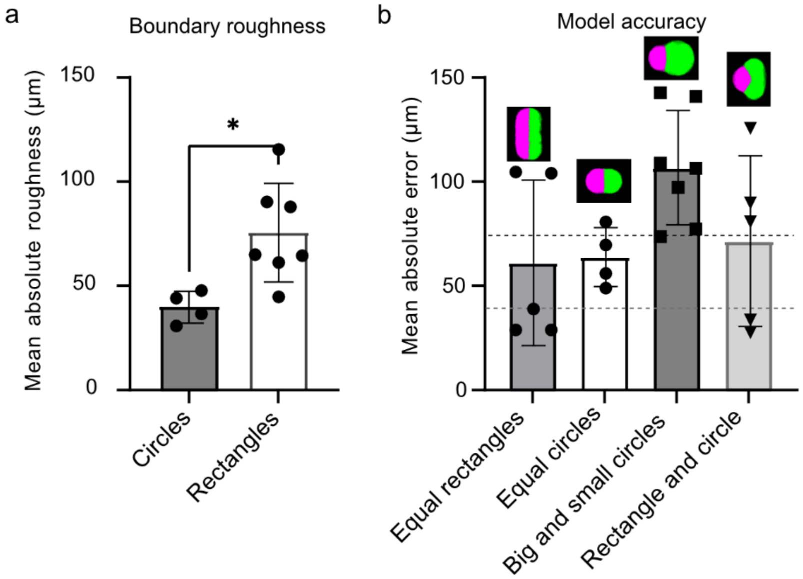

Supplementary Figure 1| Roughness and model accuracy. a, The collision boundary that forms between circles is smoother than the boundary between parallel rectangles. Roughness quantified as the mean absolute error of 2 mm line fit to the steady state interface. P value = 0.0121 using a two-sided Mann-Whitney U test, plot center is the mean, error bars are standard deviation for n=4 (circles) and n=7 (rectangles) independent tissues across three experiments. **b**, Error of model is compatible with the boundary roughness. Error was found as the mean distance from the actual boundary to the boundary in model. Black and gray dotted lines are the roughness of the boundary line between colliding rectangles and circles, respectively. Plot center is the mean, and error bars are standard deviation for n=5 (equal rectangles), n=4 (equal circles), n=7 (big and small circles), and n=5 (rectangle and circle) independent tissues across three experiments. Source data are provided as a Source Data file.

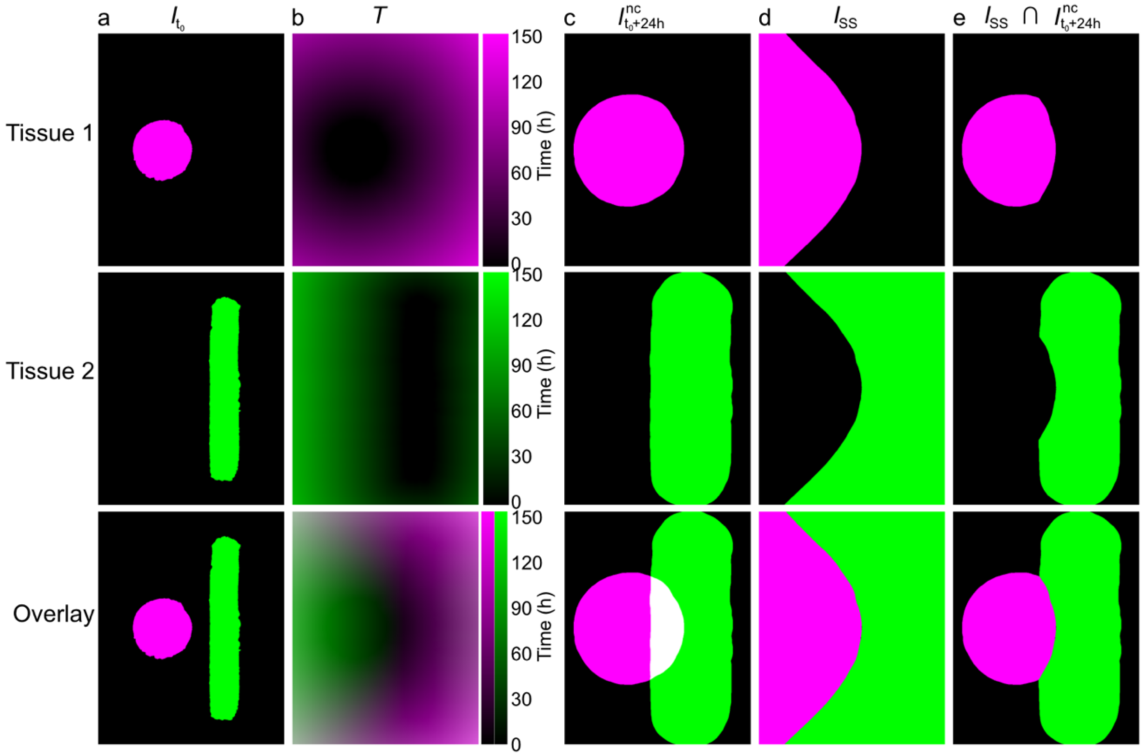

Supplementary Figure 2| Phenomenological model of collisions. a, Binary image of Initial tissue footprints (I_{t_0}). **b**, Heatmap of time that would elapse for initial tissues to fill pixels within image (T). **c**, Binary image of predicted tissue footprints after 24 h of growth, without non-mixing collision ($I^{nc}_{t_0+24h}$). **d**, Steady state forms of tissue footprints (I_{SS}), with non-mixing collisions that pin in place on contact. I_{SS} for tissue 1 is found as $T_{Tissue\ 1} < T_{Tissue\ 2}$. **e**, Binary image of predicted tissue footprints after 24 h of growth, with non-mixing collision.

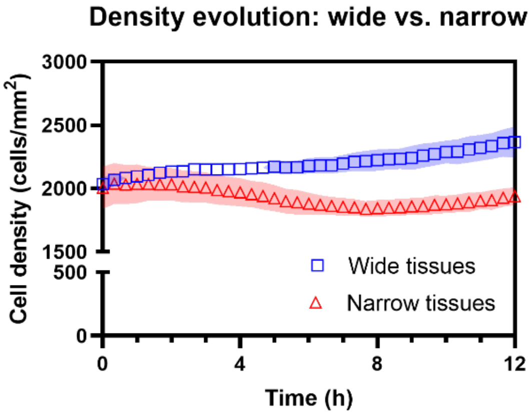

Supplementary Figure 3| Density evolution of size mismatch tissues. Although 1000 μm ('wide', blue squares) and 500 μm ('narrow', red triangles) width tissues start with the same cell density, narrower tissues expand with a lower cell density because the expansion space accounts for a larger *relative* tissue growth, consistent with previous work[1]. Densities here are calculated across the entire tissue, with shading indicating standard deviation among tissues in all five size-mismatch collisions. Source data are provided as a Source Data file.

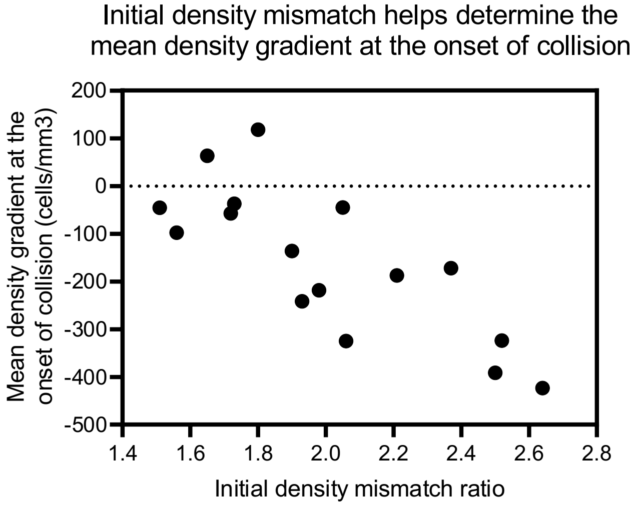

Supplementary Figure 4| Density gradient at the collision front. For tissues initially seeded at different densities, with the left tissue at a higher density than the right, the density gradient at the onset of collision is dependent on the initial density mismatch between the two tissues. The initially-denser tissues, on the left, remain denser during expansion through the onset of the collision, and hence, tissues seeded with a higher density displace those of lesser density. Source data are provided as a Source Data file.

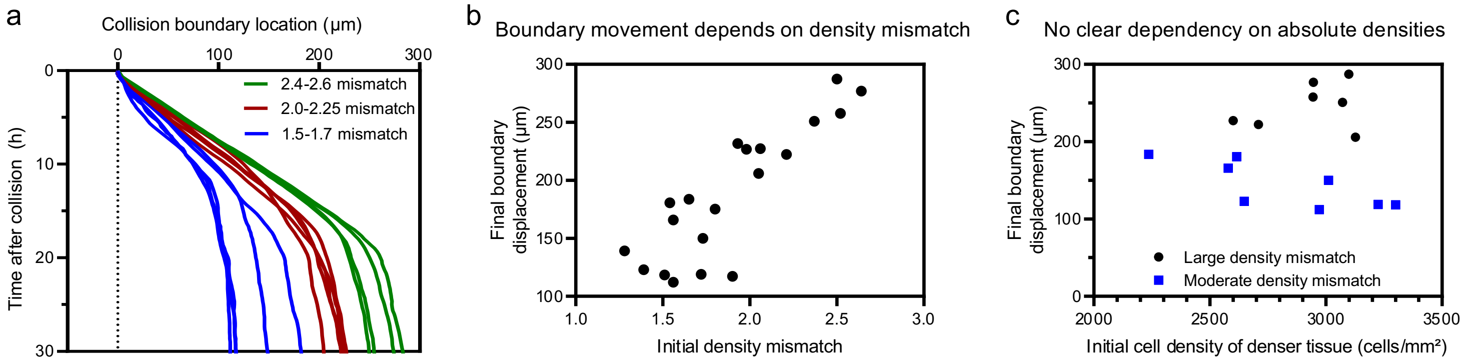

Supplementary Figure 5| Homotypic collisions at a range of initial density mismatches. a, Tissue boundary displacement over time is distinct for different initial density mismatch ratios, here shown by color for three categories of initial mismatches. **b**, Final collision boundary displacement depends on initial density mismatch ratio, ranging from 1.3 to 2.6 across all of our assay replicates, resulting in boundary displacements of 110 to 290 μm. **c**, Final boundary displacement shows no clear dependency on absolute cell densities of the colliding tissues within preserved initial density mismatch ratios. Blue squares represent an initial density ratio of 1.4-1.7; black circles represent an initial density ratio of 2.0-2.6. Source data are provided as a Source Data file.

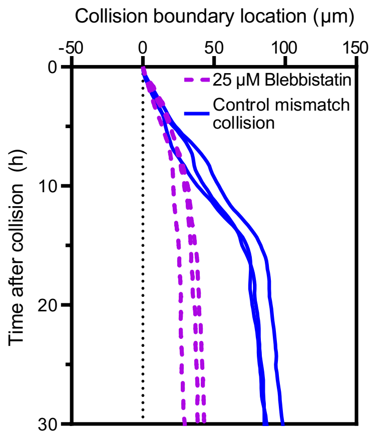

Supplementary Figure 6| Blebbistatin treatment reduces collision boundary movement. Density mismatch collisions between tissues treated with blebbistatin (purple dashed lines; see Methods) resulted in smaller boundary displacements, as compared to collisions of tissues with similar initial density mismatches (solid blue lines).

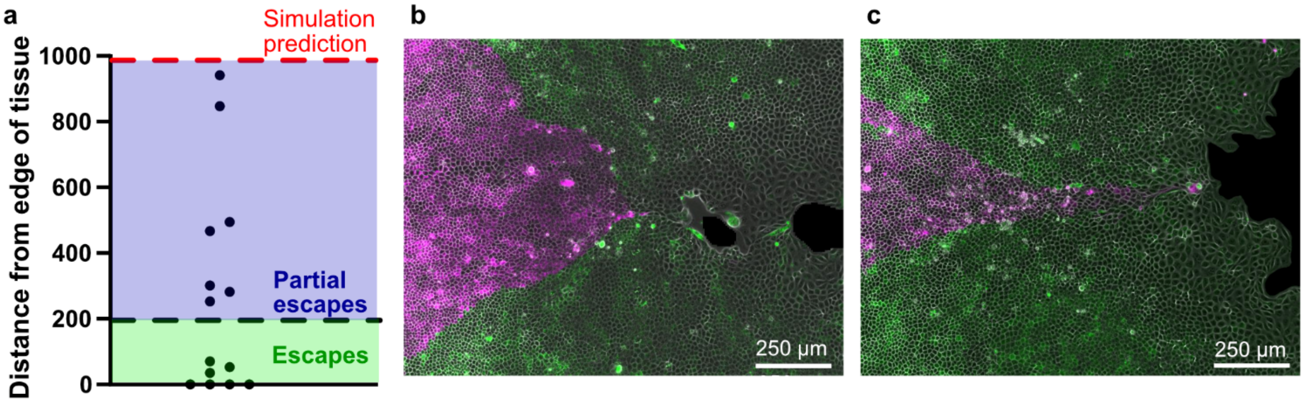

Supplementary Figure 7| Escape frequency. a, Distance of escaping tissue from the outer edge of converging tissues after 53 h of tissue growth, for converging tissues with interior angle of 110°. In these examples, the phenomenological model would indicate expectation of ~1 mm from the tissue edge at this time. Here, we denote escapes as those tissues that are within 1 correlation length of the tissue edge (~200 μm), but we note that all tissues reach closer to the edge than the simulation, indicating the escape effect is present in all tissues. **b,c** Representative escapes, with partial escape (**b**) and true escape (**c**), representative of n=8 (escape) and n=7 (no escape) tissues. Source data are provided as a Source Data file.

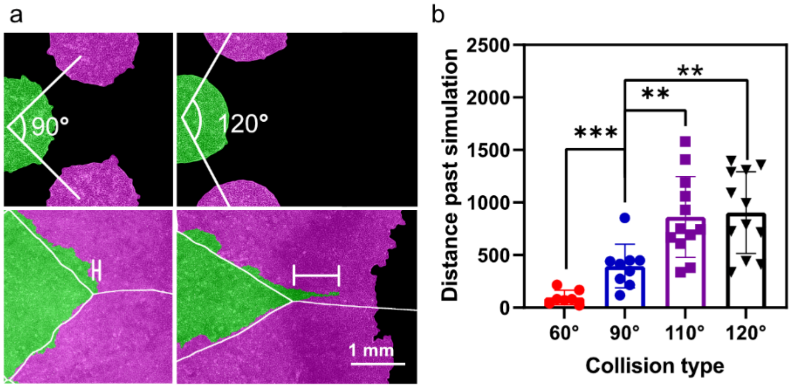

Supplementary Figure 8| Initial tissue configuration determines escape frequency. a, Representative escapes for converging tissues at an initial interior angle of 90° vs. 120°. TissEllate predicts the green tissue will migrate farther laterally between the magenta tissues in the 120° configuration than in the 90° configuration (white outlines). Even so, the green tissue exceeds these predictions by more in the 120° case. Distance bar represents the distance past simulation for these examples. **b**, Distance that escaping tissue overshoots the model at 60 h for a variety of initial tissue angles. Plot shows mean with standard deviation for n=6 (60°), n=8 (90°), n=12 (110°), and n=12 (120°) independent tissues in one experiment. P = 0.0009 (60° vs. 90°), P=0.0077 (120° vs. 90°), and P = 0.0052 (110° vs. 90°), calculated using a two-sided Mann-Whitney U test. Source data are provided as a Source Data file.

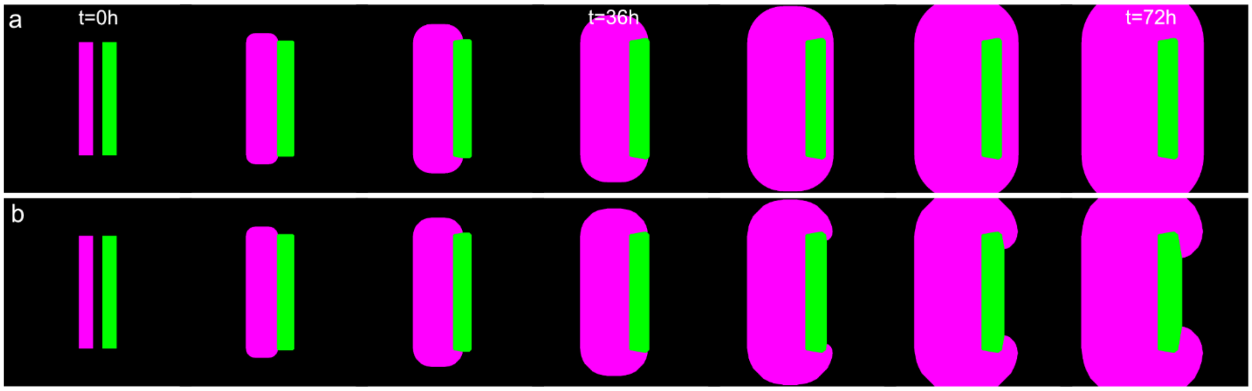

Supplementary Figure 9| Model considerations for edge speed differences and envelopment. a, Time course of envelopment for basic model, where faster tissue (magenta, 40 μm/h) unphysically grows “through” the slower tissue (green, 7 μm/h). **b**, Resetting initial tissue locations to current tissue locations periodically (here, every 6 hours) prevents unphysical envelopment. Images shown once every 12 h.

SUPPLEMENTARY NOTES

Supplementary Note 1. Model of expansion and collision of homotypic tissues

Based on our previous work[1], we modeled the expansion of monolayers of arbitrary shape by moving its boundary with constant outward normal velocity v_n. Here, we worked with pixelated binary images of initial tissue footprints I_{t_0} at time t_0 (Supplementary Fig. 2a), which enabled us to take advantage of standard image processing tools in Matlab. The goal is to find the expected tissue image footprints $I_{t_0+\Delta t}$ at any later time $t_0 + \Delta t$. For each tissue, we first used the bwdist method to calculate the distance map D, which shows the shortest distance between a given pixel and the initial tissue. This distance map is then used to calculate the time map $T = \frac{D * l_{pixel}}{v_n}$ at which a tissue reaches every pixel (Supplementary Fig. 2b), where l_{pixel} is the pixel size. In the absence of collisions with other tissues, we can then find the new tissue footprint $I_{t_0+\Delta t}^{nc}$ simply as all pixels for which $I_{time} < \Delta t$. (Supplementary Fig. 2c).

For multiple expanding tissues, we assumed that the boundary gets pinned upon collision. After a long time (steady state) each tissue thus occupies all the pixels that it manages to reach before any other tissue. The steady state tissue form I_{SS} of a given tissue thus contains all pixels for which the value of T is the minimum across all tissues (Supplementary Fig. 2d). Note that for the homotypic tissues that expand with equal outward speed v_n, the steady state tissue form I_{SS} corresponds to the pixels that are the closest to a given tissue. The time evolution of each tissue image $I_{t_0+\Delta t}$ can then be obtained as the intersection between the tissue footprint without collisions $I_{t_0+\Delta t}^{nc}$ and the steady state tissue form I_{SS}, i.e., $I_{t_0+\Delta t} = I_{t_0+\Delta t}^{nc} \cap I_{SS}$ (Supplementary Fig. 2e).

Supplementary Note 2. Model extension for heterotypic tissues

The model described above has to be slightly adjusted for the heterotypic tissues that expand with different outward velocities. The problem is that the model above predicts that a faster expanding tissue migrates "through" a slower expanding tissue to reach distant points (Supplementary Fig. 9a). To correct for this issue, we repeatedly calculate the distance map D with respect to the current tissue forms (rather than initial forms) for all tissues at a time interval

chosen such that the slower tissue expands by at least 4 pixels and the faster tissue expands by no more than half the width of the slower tissue. A smaller pixel size may be used to address any time interval or resolution issues. The rest of the steps are identical as in the model described above. This slight modification of the model is sufficient to properly capture how faster expanding tissues envelop slower ones (Supplementary Fig. 9b).

References

1. Heinrich, M. A., Alert, R., Lachance, J. M., Zajdel, T. J., Kosmrlj, A., & Cohen, D. J. (2020). Size-dependent patterns of cell proliferation and migration in freely-expanding epithelia. *eLife*, *9*, e58945. https://doi.org/10.7554/ELIFE.58945
