# Supplementary material for: Self-assembly of tessellated tissue sheets by expansion and collision
Source: Nat Commun. 2022 Jul 12;13:4026. doi: 10.1038/s41467-022-31459-1 (PMC9276766; doi:10.1038/s41467-022-31459-1)
Supplement: Supplementary file 3 — Description of additional Supplementary Files [file 41467_2022_31459_MOESM3_ESM.pdf]

Descriptions of Additional Supplementary Data Files

Supplementary Video 1| Collisions of archetypal shapes. From left to right: rectangle pairs, circular pairs, circle and larger circle, and circle and rectangle collisions. Fluorescence channels were segmented and overlaid with phase channel (Methods). Movies includes 60 h.

Supplementary Video 2| Rectangle collision nonmixing dynamics. Fluorescence channels and phase overlay (left) clearly shows the boundary is sharp with no mixing. This makes segmentation of fluorescence channels appropriate for visualization (right, see methods for details). Boundaries between rectangle initial tissues form by colliding all along the collision line at once.

Supplementary Video 3| Circle collision dynamics. Boundaries between initial circular tissues form from a small collision region that then extends outward.

Supplementary Video 4| Collisions of archetypal shapes with model. Model accurately reproduces collision dynamics, with less accuracy at sharp corners of rectangular tissues. Yellow and blue lines represent the model prediction for green and magenta tissues, respectively.

Supplementary Video 5| Dynamics of collision boundary- MDCK width or density mismatch. On top, a control collision: a rectangular pair matched in both size and cell density features oscillating dynamics with little bias in boundary motion. In the middle, a width mismatch: a rectangular pair mismatched in tissue width but matched in cell density features translation of the boundary away from the wider tissue (1000 μm) toward the narrower tissue (500 μm). On the bottom, a density mismatch: a rectangular pair mismatched in cell density but matched in tissue size features translation of the boundary away from the high density tissue (~2600 cells/mm^2) toward the low density tissue (~1800 cells/mm^2). All fluorescent channels were segmented for visualization.

Supplementary Video 6| Dynamics of collision boundary- range of initial density mismatches. Rectangular pair mismatched in cell density but matched in tissue size features translation of the boundary away from the high density tissue toward the low density tissue, here shown for a range of initial density mismatch ratios—1.5 (top), 2.0 (middle), and 2.5 (bottom). In all three collisions, the denser tissue is presented in green on the left side. Fluorescent channels were segmented for visualization.

Supplementary Video 7| Dynamics of collision boundary- translation reduced by blebbistatin treatment. Density mismatch collisions between tissues treated with blebbistatin (bottom; see Methods) results in smaller translation of the boundary, as compared to collisions of tissues with similar initial density mismatches (top). Fluorescent channels were segmented for visualization.

Supplementary Video 8| TissEllate- MDCK Escher. Three color collision dynamics of pattern from Fig. 3A-D.

Supplementary Video 9| TissEllate- MDCK circle tessellation. Two color collision dynamics of tissues (filled color) vs. predicted model steady-state tissue boundaries (white lines) for hexagonal lattice tessellation of circular monolayers.

Supplementary Video 10| TissEllate- MDCK rectangle tessellation. Two color collision dynamics of tissues (filled color) vs. predicted model steady-state tissue boundaries (white lines) for tessellation of rectangular monolayers.

Supplementary Video 11| 'Escape.' One tissue migrating between two converging tissues forms a long, necked down region that we denote an "escape."

Supplementary Video 12| Minimal escape. When the converging tissues collide ahead of the escaping tissue, very little necking down can occur.

Supplementary Video 13| Dynamics of heterotypic collision boundary- MCF10A control. MCF10A monolayer pairs feature similar nonmixing dynamics as MDCK monolayers.

Supplementary Video 14| Dynamics of heterotypic collision boundary- MCF10A with MDA-MB-231. MCF10A monolayer (left) displaces MDA-MB-231 monolayer (right).

Supplementary Video 15| Dynamics of heterotypic collision boundary- MCF10A with MCF7. MCF10A monolayer (left) is faster than MCF7 monolayer (right), but is displaced backward at the collision.

Supplementary Video 16| Engulfment of MCF7 monolayer by MCF10A monolayer. MCF10A monolayer (rectangle) is faster than MCF7 monolayer (circle), and therefore grows around the MCF7 monolayer until total engulfment.
