# Supplementary material for: Self-assembly of tessellated tissue sheets by expansion and collision
Source: Nat Commun. 2022 Jul 12;13:4026. doi: 10.1038/s41467-022-31459-1 (PMC9276766; doi:10.1038/s41467-022-31459-1)
Supplement: Supplementary file 20 — Reporting Summary [file 41467_2022_31459_MOESM20_ESM.pdf]

nature research

Corresponding author(s): Dr. Andrej Košmrlj (andrej@princeton.edu) and Dr. Daniel J. Cohen (danielcohen@princeton.edu)

Last updated by author(s): Dec 23, 2021

Reporting Summary

Nature Research wishes to improve the reproducibility of the work that we publish. This form provides structure for consistency and transparency in reporting. For further information on Nature Research policies, see our Editorial Policies and the Editorial Policy Checklist.

Statistics

For all statistical analyses, confirm that the following items are present in the figure legend, table legend, main text, or Methods section.

n/a	Confirmed	
☐	☒	The exact sample size (*n*) for each experimental group/condition, given as a discrete number and unit of measurement
☐	☒	A statement on whether measurements were taken from distinct samples or whether the same sample was measured repeatedly
☐	☒	The statistical test(s) used AND whether they are one- or two-sided<br>*Only common tests should be described solely by name; describe more complex techniques in the Methods section.*
☒	☐	A description of all covariates tested
☒	☐	A description of any assumptions or corrections, such as tests of normality and adjustment for multiple comparisons
☐	☒	A full description of the statistical parameters including central tendency (e.g. means) or other basic estimates (e.g. regression coefficient) AND variation (e.g. standard deviation) or associated estimates of uncertainty (e.g. confidence intervals)
☐	☒	For null hypothesis testing, the test statistic (e.g. *F*, *t*, *r*) with confidence intervals, effect sizes, degrees of freedom and *P* value noted<br>*Give P values as exact values whenever suitable.*
☒	☐	For Bayesian analysis, information on the choice of priors and Markov chain Monte Carlo settings
☒	☐	For hierarchical and complex designs, identification of the appropriate level for tests and full reporting of outcomes
☒	☐	Estimates of effect sizes (e.g. Cohen's *d*, Pearson's *r*), indicating how they were calculated

Our web collection on statistics for biologists contains articles on many of the points above.

Software and code

Policy information about availability of computer code

Data collection	NIS Elements software (Nikon, v.5.20.01) was used in the imaging of the data for this study.
Data analysis	We used MATLAB R2018b (Mathworks) and ImageJ (NIH, v.2.35) for all data analysis, with procedures and custom codes as described in the text of the manuscript. Statistical tests were performed using GraphPad Prism version 9.1.0. Key code used for analysis in this study is available on our laboratory repository: https://github.com/CohenLabPrinceton/TissEllation.git

For manuscripts utilizing custom algorithms or software that are central to the research but not yet described in published literature, software must be made available to editors and reviewers. We strongly encourage code deposition in a community repository (e.g. GitHub). See the Nature Research guidelines for submitting code & software for further information.

Data

Policy information about availability of data

All manuscripts must include a data availability statement. This statement should provide the following information, where applicable:

- Accession codes, unique identifiers, or web links for publicly available datasets
- A list of figures that have associated raw data
- A description of any restrictions on data availability

The complete raw data generated during the current study are available from the corresponding authors upon reasonable request due to the size of the datasets (terabytes). Representative data and analysis code can be found at https://github.com/CohenLabPrinceton/TissEllation.git

Field-specific reporting

Please select the one below that is the best fit for your research. If you are not sure, read the appropriate sections before making your selection.

☒ Life sciences ☐ Behavioural & social sciences ☐ Ecological, evolutionary & environmental sciences

For a reference copy of the document with all sections, see nature.com/documents/nr-reporting-summary-flat.pdf

Life sciences study design

All studies must disclose on these points even when the disclosure is negative.

Sample size	Sample included >= 4 replicates (discrete tissues or tissue arrays), across at least 3 independently-performed experiments to ensure reproducibility. No calculations were needed to determine appropriate sample sizes.
Data exclusions	In data for Fig. 2e,f,h, any tissues that migrated >30% slower than the expected migration rate of 30 um/h, caused by matrix rip-off during stencil removal, were excluded. This criterion was determined before the experiments were performed and resulted in 3 exclusions for Fig. 2e,f and another 2 exclusions for Fig. S5 (a component of Fig. 2h). No other samples were excluded in the study.
Replication	All assays were completed with at least 3 independent experiments on different days to ensure reproducibility. Several assays were also verified through repetition by different individuals. All experimental details were described in our Methods section.
Randomization	Randomization was not relevant to this study as our analyses and conclusions did not depend on statistical significance.
Blinding	Blinding was not relevant to the study as analyses were automated and performed equally for all replicates thereby eliminating bias

Reporting for specific materials, systems and methods

We require information from authors about some types of materials, experimental systems and methods used in many studies. Here, indicate whether each material, system or method listed is relevant to your study. If you are not sure if a list item applies to your research, read the appropriate section before selecting a response.

Materials & experimental systems

n/a	Involved in the study
☒	☐ Antibodies
☐	☒ Eukaryotic cell lines
☒	☐ Palaeontology and archaeology
☒	☐ Animals and other organisms
☒	☐ Human research participants
☒	☐ Clinical data
☒	☐ Dual use research of concern

Methods

n/a	Involved in the study
☒	☐ ChIP-seq
☒	☐ Flow cytometry
☒	☐ MRI-based neuroimaging

Eukaryotic cell lines

Policy information about cell lines

Cell line source(s)	MDCK-II cell line: gift from the James Nelson lab, Stanford, CA. MCF10A, MCF7, MDA-MB-231 cell lines: a gift from the Celeste Nelson lab, Princeton, NJ, and commercially available at ATCC.
Authentication	None of the cell lines used were authenticated.
Mycoplasma contamination	All cells were thawed from previously frozen cryostocks that were tested and certified to be negative for mycoplasma at the time of cryopreservation.
Commonly misidentified lines (See ICLAC register)	No cell lines used are commonly misidentified.
